# The Acid–Base Effects of Albumin in Sepsis: Reconciling the Stewart Physicochemical Approach with the Traditional Buffer–Base Paradigm

**DOI:** 10.3390/jcm15155789

**Published:** 2026-07-24

**Authors:** Daniele Orso, Raffaele Saro, Giorgio Della Rocca

**Affiliations:** 1 Department of Anesthesia and Intensive Care Medicine, “Santa Maria della Misericordia” Udine University Hospital, Azienda Sanitaria Universitaria Friuli Centrale (ASUFC), 33100 Udine, Italy; 2 Department of Medicine (DMED), University of Udine, 33100 Udine, Italy; raffaele.saro@spes.uniud.it (R.S.); giorgio.dellarocca@uniud.it (G.D.R.)

**Keywords:** albumin, Stewart approach, acid–base physiology, sepsis, strong ion difference, critical care, hypoalbuminemia

## Abstract

Albumin administration in sepsis and septic shock remains controversial because of uncertain clinical benefits and complex acid–base effects. Unlike crystalloids, albumin influences acid–base equilibrium through effects on chloride balance, strong ion difference (SID), buffering systems, and weak acid concentration. These effects may be interpreted differently by the traditional bicarbonate-centered framework and Stewart’s physicochemical approach. In the traditional paradigm, albumin acts mainly as a non-bicarbonate plasma buffer influencing base excess and anion gap interpretation. In the Stewart framework, albumin is a weak non-volatile acid contributing to total weak acids (Atot), thereby influencing hydrogen ion dissociation and pH regulation. This narrative review compares these two approaches to albumin-related acid–base physiology in sepsis, with emphasis on chloride balance, strong ion difference, hypoalbuminemia, dilutional effects, and cardiorespiratory interactions during mechanical ventilation and septic shock. We also contextualize these mechanisms within major randomized trials of albumin and resuscitation fluids and present illustrative conceptual simulations illustrating the directional acid–base effects of different fluid compositions. The narrative review focuses on masked acidosis in hypoalbuminemic patients and on the interpretation of pH changes after albumin administration. Finally, we propose an integrated bedside framework combining traditional, physicochemical, respiratory, and hemodynamic variables for acid–base interpretation during fluid resuscitation.

## 1. Introduction

Fluid resuscitation remains one of the cornerstones of sepsis management [1,2,3]. However, the physiological consequences of different resuscitation fluids remain incompletely understood, particularly regarding acid–base equilibrium. Current international sepsis guidelines recommend balanced crystalloids as the preferred first-line resuscitation fluid, whereas albumin may be considered in patients requiring substantial crystalloid volumes [1]. Despite these recommendations, interpretation of albumin-associated acid–base changes remains challenging in daily clinical practice because reductions in pH after albumin administration may be misinterpreted as worsening metabolic acidosis rather than restoration of weak-acid balance. Although crystalloids have traditionally dominated initial resuscitation strategies, albumin continues to attract interest because of its unique physiological properties and its potential role in severe sepsis and septic shock [4,5,6].

Albumin differs fundamentally from crystalloid solutions because it simultaneously influences oncotic pressure, endothelial function, drug transport, antioxidant activity, intravascular volume, and acid–base physiology [7,8,9]. Unlike balanced crystalloids or saline, albumin administration modifies both strong ion balance and the circulating concentration of weak acids, producing acid–base effects that are often difficult to interpret at the bedside.

Traditionally, acid–base disorders have been interpreted using the Henderson–Hasselbalch equation and the bicarbonate-centered framework, in which albumin acts primarily as a non-bicarbonate plasma buffer influencing buffering capacity, base excess, and anion gap interpretation [10]. In contrast, Stewart proposed a physicochemical model in which pH is determined by three independent variables—partial pressure of carbon dioxide (PaCO_2_), strong ion difference (SID), and the total concentration of weak acids (Atot)—with albumin acting as a major weak non-volatile acid directly contributing to Atot.

Although these two frameworks are often presented as competing approaches, they provide complementary perspectives on the same physiological processes. Their integration becomes particularly relevant in septic patients, in whom hypoalbuminemia, hyperchloremia, hyperlactatemia, dilutional effects, and respiratory disturbances frequently coexist. Under these conditions, a reduction in pH after albumin administration may reflect restoration of weak acids rather than worsening tissue hypoperfusion, whereas severe hypoalbuminemia may partially mask clinically important metabolic acidosis.

Several reviews have addressed either Stewart acid-base physiology or the clinical use of albumin in critically ill patients [11,12,13]. However, few have specifically integrated the traditional buffer–base paradigm with the physicochemical approach while placing these concepts into the context of contemporary sepsis resuscitation, randomized clinical trials, and practical bedside interpretation.

The aim of this narrative review is to reconcile traditional and physicochemical interpretations of albumin-related acid–base physiology in sepsis. We integrate mechanistic concepts, evidence from major randomized trials, illustrative conceptual simulations, and bedside implications to propose a practical framework for interpreting acid–base changes during albumin administration. This article is intended as a mechanistic and clinically oriented narrative review rather than as a systematic evidence synthesis.

## 2. Methods for Narrative Review

This narrative review was conducted to provide a mechanistic and clinically oriented overview of the acid–base effects of albumin administration in patients with sepsis and septic shock. The review was designed to integrate the traditional buffer–base paradigm with Stewart’s physicochemical approach while placing these concepts into the context of contemporary fluid resuscitation and critical care practice. The review question and objectives were defined a priori.

A literature search was performed in PubMed/MEDLINE, Embase, and Scopus from database inception through March 2026 using combinations of the following keywords: albumin, acid-base, Stewart, strong ion difference, SID, Atot, weak acids, physicochemical, buffer base, base excess, anion gap, hyperchloremia, fluid resuscitation, sepsis, septic shock, and critical care. Reference lists of relevant articles were also manually screened to identify additional eligible publications.

Priority was given to landmark physiological studies describing acid–base mechanisms, methodological papers on the Stewart approach, clinical investigations of albumin-related acid–base effects, randomized controlled trials evaluating albumin or resuscitation fluids, and contemporary international guidelines relevant to sepsis management. More recent publications were preferentially selected when multiple studies addressed similar concepts.

Because this was a narrative review, no formal systematic study selection process, quantitative evidence synthesis, or risk-of-bias assessment was performed. The final selection of references was based on their methodological quality, historical relevance, physiological significance, and clinical applicability to the interpretation of albumin-associated acid–base disturbances.

This review was prepared in accordance with the principles proposed by the Scale for the Assessment of Narrative Review Articles (SANRA), with particular attention to transparency of literature identification, balanced interpretation of evidence, and clear definition of the review objectives [14]. The review was conducted in accordance with current recommendations for conducting and reporting narrative reviews while recognizing that narrative reviews are intended to provide conceptual integration rather than exhaustive systematic evidence synthesis. ChatGPT (ver. GPT 5.5, OpenAI, San Francisco, CA, USA) was used to assist in the preparation of the graphical figures and for English language editing. The authors independently verified all scientific content and are solely responsible for the final manuscript.

## 3. Historical Evolution of Acid–Base Interpretation

Modern acid–base physiology emerged through progressive conceptual refinement. Early physiological models focused primarily on the relationship between carbon dioxide and bicarbonate, whereas later approaches attempted to integrate electrolyte balance, buffering systems, and physicochemical principles into a unified interpretation of plasma acid–base equilibrium.

The Henderson equation, introduced in the early twentieth century and later reformulated by Hasselbalch into logarithmic form, established the conceptual basis of bicarbonate-centered acid–base analysis [10,15]. Within this framework, pH was interpreted as a function of bicarbonate concentration and dissolved carbon dioxide tension. This framework rapidly became the dominant clinical approach because it was intuitive, mathematically accessible, and closely aligned with arterial blood gas analysis.

Subsequent work by Van Slyke and Siggaard-Andersen extended the bicarbonate-centered framework through the concepts of buffer base and base excess, improving its clinical applicability [16,17,18].

Despite its clinical usefulness, the traditional bicarbonate-centered framework encountered increasing limitations in critically ill patients characterized by mixed electrolyte abnormalities, dilutional states, hypoalbuminemia, hyperchloremia, and large-volume fluid resuscitation. These limitations highlighted the need for a more mechanistic interpretation of acid–base physiology, which was provided by Stewart’s physicochemical model [19,20]. Stewart proposed a physicochemical approach in which hydrogen ion concentration was determined by three independent variables: PaCO_2_, strong ion difference (SID), and total concentration of weak acids (Atot) [20]. In this framework, bicarbonate was no longer considered an independent regulator of pH but rather a dependent consequence of physicochemical equilibrium.

Fencl and colleagues later translated Stewart’s mathematically complex model into a clinically accessible bedside approach by introducing simplified calculations for SID, albumin effects, and unmeasured anions [21,22,23]. This adaptation substantially increased the clinical applicability of physicochemical acid-base interpretation in critical care medicine.

Although Stewart introduced his physicochemical framework in the late 1970s and early 1980s, widespread clinical recognition of saline-induced hyperchloremic metabolic acidosis became widely recognized only later, particularly following experimental and clinical studies published during the late 1990s and early 2000s [24,25]. Within this historical context, albumin occupies a particularly important conceptual position because it lies at the intersection of the two frameworks. In the traditional model, albumin is viewed primarily as a plasma buffer influencing base excess and anion gap interpretation. In the Stewart framework, albumin becomes one of the principal determinants of Atot and therefore a direct regulator of acid–base equilibrium.

The evolution from traditional to physicochemical interpretation should not be regarded as a replacement of one framework by the other. Rather, the Stewart approach expanded mechanistic understanding of acid-base physiology while traditional variables retained substantial practical and prognostic utility. Contemporary critical care practice increasingly relies on integration of both perspectives rather than exclusive adherence to either framework. This integrated perspective provides the conceptual basis for the present review.

## 4. The Traditional Buffer–Base Interpretation

The traditional approach to acid–base physiology is centered on the Henderson–Hasselbalch equation:

pH = 6.1 + log(HCO_3_^−^/(0.03 × PaCO_2_))

Within this framework, pH is determined by the relationship between bicarbonate concentration and PaCO_2_ [15].

Albumin functions primarily as a non-bicarbonate plasma buffer because of its multiple negatively charged residues capable of binding hydrogen ions. Consequently, hypoalbuminemia reduces total plasma buffering capacity.

This phenomenon has several clinically important consequences. First, hypoalbuminemia lowers the measured anion gap despite persistent accumulation of unmeasured anions [26,27]. Correction formulas for albumin-adjusted anion gap are therefore recommended in critically ill patients. Second, severe hypoalbuminemia reduces plasma buffering capacity in the traditional framework, while in the Stewart model the associated reduction in total weak acids (Atot) exerts an alkalinizing tendency. These represent complementary but conceptually distinct physiological mechanisms. Conversely, albumin administration may appear acidifying because restoration of weak acid concentration may lower bicarbonate concentration and base excess despite stable or improving tissue perfusion [28,29,30,31]. However, the traditional framework often struggles to distinguish albumin-specific effects from concomitant changes in chloride balance, sodium concentration, dilution, renal compensation, and tissue perfusion.

## 5. The Stewart Physicochemical Approach

Stewart proposed that hydrogen ion concentration is determined by three independent variables:

- Partial pressure of carbon dioxide (PaCO_2_)
- Strong ion difference (SID)
- Total concentration of weak acids (Atot)

This framework may be summarized conceptually as:

pH = f(PaCO_2_, SID, A_tot_)

Within plasma, albumin and phosphate constitute the principal weak acids contributing to Atot. Albumin behaves as a polyprotic weak acid with an apparent pKa close to physiological pH. Consequently, its net negative charge is not fixed but varies continuously according to ambient pH. As pH increases, albumin becomes progressively more negatively charged through dissociation of weak acidic groups, whereas lower pH promotes protonation and reduces its net charge. This pH-dependent behavior explains why albumin contributes dynamically to Atot rather than acting as a passive buffer and provides the physiological basis for the simplified Figge–Fencl equations used in clinical practice.

Strong ion difference may be simplified as:

SID = [Na^+^] + [K^+^] + [Ca^2+^] + [Mg^2+^] − [Cl^−^] − [Lactate^−^]

When measured ions cannot fully account for the observed acid–base disturbance, the Strong Ion Gap (SIG) provides an estimate of unmeasured strong anions. Increased SIG values suggest accumulation of unmeasured acids (e.g., ketoacids or uremic anions) and may help explain persistent metabolic acidosis despite apparently normal measured electrolytes.

According to Stewart physiology, increases in Atot favor hydrogen ion dissociation and therefore lower pH [20]. Conversely, reductions in Atot exert an alkalinizing tendency. Fluids with SID substantially lower than plasma SID produce metabolic acidosis, whereas fluids with higher SID are relatively alkalinizing. Albumin administration may influence acid–base equilibrium through at least two simultaneous mechanisms:

- increased Atot (weak acid effect);
- altered SID due to accompanying chloride load.

This distinction explains why apparently similar acid–base changes may emerge through fundamentally different mechanisms. Interpretation of albumin administration should consider changes in Atot and SID simultaneously rather than relying exclusively on bicarbonate or base excess.

## 6. Traditional Versus Stewart Interpretations

**Figure 1:**
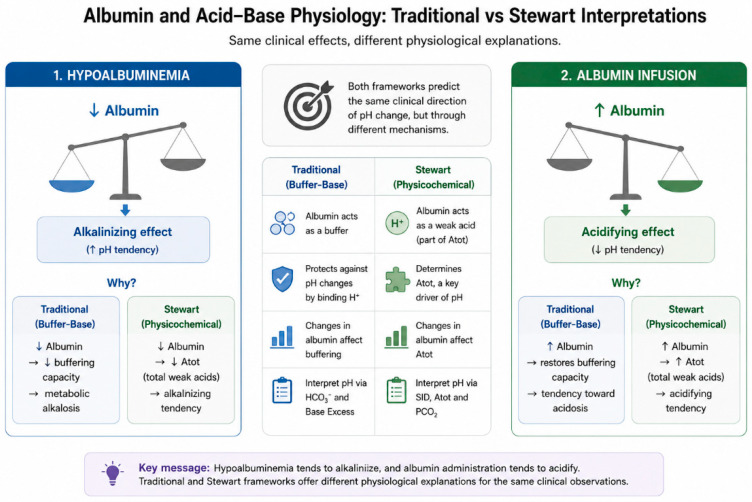
Comparison between the traditional buffer–base interpretation and the Stewart physicochemical approach to albumin-related acid-base physiology. According to the traditional acid–base model, hypoalbuminemia reduces plasma buffering capacity, whereas hyperalbuminemia increases it. In Stewart’s physicochemical framework, hypoalbuminemia decreases the total concentration of non-volatile weak acids (Atot), while an increase in albumin concentration results in a corresponding increase in Atot.

The traditional and Stewart approaches are often presented as alternative or competing models of acid–base physiology. In the context of albumin administration, however, they are better regarded as complementary interpretative frameworks because they describe the same biological system from different perspectives [32]. Whereas the traditional approach focuses on bicarbonate and buffer-base relationships, the Stewart model emphasizes the physicochemical determinants of acid–base equilibrium. The principal conceptual differences between the two frameworks are summarized in Figure 1.

The Stewart approach shifts the interpretative focus from bicarbonate to physicochemical variables [22,23]. In this model, bicarbonate is a dependent rather than an independent determinant of pH, resulting from the interaction among PaCO_2_, SID, and Atot. Albumin therefore functions not only as a plasma buffer but also as one of the principal weak acids contributing to Atot. Consequently, hypoalbuminemia reduces Atot and exerts an alkalinizing tendency, whereas albumin administration increases Atot and may lower pH.

This distinction is particularly relevant in septic shock, where hypoalbuminemia, hyperchloremia, hyperlactatemia, renal dysfunction, and large-volume fluid administration frequently coexist. In these circumstances, the traditional framework accurately identifies the presence of metabolic acidosis but may not fully explain changes in bicarbonate or base excess following albumin infusion. Conversely, the Stewart approach provides a mechanistic explanation for these changes, although it is generally less intuitive for routine bedside use [33].

Neither framework alone implies clinical deterioration. A modest reduction in pH after albumin administration may occur despite improving hemodynamics because it reflects restoration of weak acid concentration rather than worsening tissue hypoperfusion. Integrating the traditional and physicochemical approaches therefore provides the most informative interpretation of albumin-associated acid–base changes in sepsis. The main conceptual differences between the two frameworks are also summarized in Table 1.

## 7. Albumin Charge, Weak Acids, and the Figge–Fencl Bridge

Albumin is not simply a passive plasma buffer but a polyprotic weak acid whose net electrical charge varies dynamically with pH. Multiple histidine, carboxyl, and amino acid side chains undergo protonation and deprotonation within the physiological pH range, making albumin the principal non-volatile weak acid in plasma. Consequently, the effective negative charge carried by albumin is not fixed but increases as pH rises and decreases as pH falls. This pH-dependent behavior explains why albumin contributes dynamically to acid–base equilibrium rather than acting as an inert buffer.

In Stewart’s physicochemical model, these properties are summarized by the variable Atot, representing the total concentration of weak acids, of which albumin accounts for the largest fraction under physiological conditions. Thus, hypoalbuminemia reduces Atot and exerts an alkalinizing tendency, whereas albumin administration increases Atot and may lower pH by restoring the concentration of weak acids. Importantly, these effects arise from changes in weak-acid concentration rather than from alterations in bicarbonate itself.

The work of Figge, Fencl, and colleagues provided the practical bridge between Stewart’s physicochemical theory and routine bedside interpretation by quantitatively describing the pH-dependent electrical charge of albumin and incorporating this behavior into clinically applicable acid–base calculations [23]. Their work also clarified why hypoalbuminemia lowers the measured anion gap independently of changes in unmeasured anions.

Consequently, hypoalbuminemic patients may appear to have a normal anion gap despite clinically relevant accumulation of unmeasured acids. A commonly used bedside correction is:

Corrected AG = measured AG + 2.5 × (4.0 − albumin [g/dL])

For example, a patient with sepsis, albumin 2.0 g/dL, and a measured anion gap of 12 mEq/L would have a corrected anion gap of: 12 + 2.5 × (4.0 − 2.0) = 17 mEq/L. Because hypoalbuminemia lowers the measured AG independently of unmeasured anions, albumin-corrected AG may facilitate bedside interpretation in selected patients, although this correction remains an approximation and should be interpreted together with the overall clinical and physicochemical context.

Thus, an apparently normal anion gap may conceal a clinically relevant accumulation of unmeasured anions. In Stewart terms, the same phenomenon can be interpreted as the alkalinizing effect of reduced Atot partially offsetting the acidifying effect of lactate, chloride, or other unmeasured anions. Reduced buffering capacity and alkalinizing tendency should therefore be regarded as complementary but physiologically distinct consequences of hypoalbuminemia: the former reflects diminished capacity to buffer additional acid loads, whereas the latter reflects the equilibrium effect of reduced weak acid concentration on plasma pH.

## 8. Physicochemical Simulations of Albumin Administration

**Figure 2:**
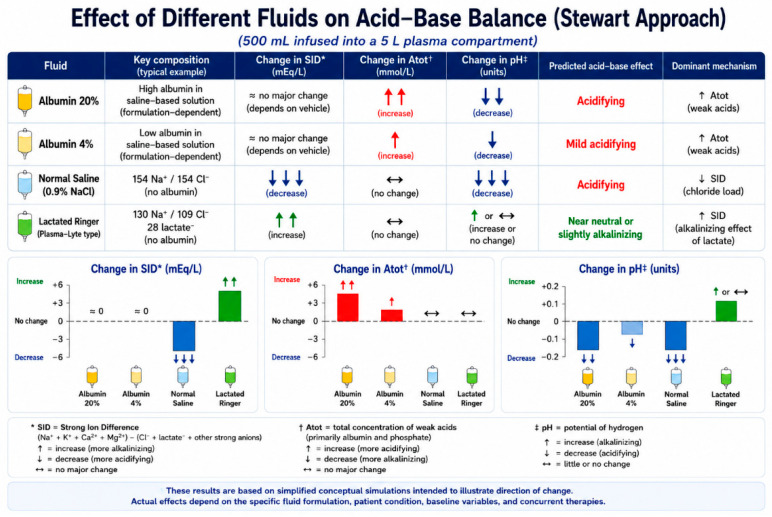
Conceptual physicochemical simulations comparing albumin 20%, albumin 4%, normal saline, and lactated Ringer solution. Illustrative conceptual simulations based on simplified Stewart principles; values are intended to demonstrate directional trends rather than calibrated physiological predictions.

To illustrate the physiological mechanisms discussed in this review, we developed simplified conceptual simulations based on Stewart’s physicochemical framework under predefined theoretical conditions. These simulations were designed exclusively to demonstrate the expected direction of acid–base changes resulting from variations in albumin concentration, carrier electrolyte composition, strong ion difference (SID), chloride concentration, and lactate, rather than to provide calibrated patient-level predictions. Figure 2 compares the conceptual physicochemical effects of albumin 20%, albumin 4%, normal saline, and lactated Ringer solution under the predefined assumptions described in the Supplementary Methods.

The simulations illustrate three complementary concepts. First, they compare the expected physicochemical effects of common resuscitation fluids with different electrolyte composition and weak acid content. Second, they distinguish SID-mediated acidification, which predominates with saline, from Atot-mediated changes associated with albumin administration. Third, they demonstrate how hypoalbuminemia may partially mask an underlying metabolic acidosis and how restoration of albumin concentration may reveal the same metabolic disturbance without implying clinical deterioration.

These simulations are intended exclusively as conceptual illustrations of Stewart physiology and should not be interpreted as validated quantitative models, predictors of individual patient responses, or representations of any specific commercial albumin formulation.

## 9. Fluid Composition, Chloride Burden, and Strong Ion Difference

The relationship between chloride balance and acid–base physiology represents one of the most clinically relevant applications of Stewart’s physicochemical framework. Among critically ill patients, hyperchloremia is common and frequently results from administration of chloride-rich intravenous fluids, particularly normal saline and some albumin preparations [31,33].

Within the traditional framework, saline-associated metabolic acidosis has historically been described as “dilutional” or “hyperchloremic” acidosis. However, Stewart physiology provides a mechanistic explanation by demonstrating that chloride excess reduces strong ion difference (SID), thereby favoring hydrogen ion dissociation and lowering pH [29].

Normal plasma SID is approximately 38–42 mEq/L, whereas the SID of normal saline is effectively zero because sodium and chloride concentrations are equal. Administration of large volumes of saline therefore lowers plasma SID and predictably produces metabolic acidosis. Balanced crystalloids differ substantially in this regard. Solutions such as lactated Ringer or Plasma-Lyte contain lower chloride concentrations and higher effective SID values. Consequently, they exert smaller acidifying effects compared with saline-based fluids.

This distinction has become increasingly relevant following major randomized trials comparing balanced crystalloids and saline. Trials such as SPLIT, SMART, BaSICS, and PLUS primarily evaluated clinical outcomes rather than acid–base physiology, yet they collectively reinforced the concept that fluid composition itself may influence renal function, electrolyte balance, and acid–base equilibrium [34,35,36,37,38]. The acid–base effects of intravenous fluids depend not only on the administered volume but also on their electrolyte composition and effective SID.

Although the magnitude of clinical benefit remains debated, several mechanistic pathways have been proposed through which chloride-rich solutions may adversely affect organ function. Experimental studies suggest that hyperchloremia may induce renal vasoconstriction through tubuloglomerular feedback mechanisms, potentially reducing renal cortical perfusion and glomerular filtration [39]. Hyperchloremia may also influence inflammatory signaling, endothelial function, and microcirculatory regulation [33,39].

Albumin solutions require additional interpretation because they modify weak acid concentration and, depending on the specific commercial formulation, may also influence strong ion difference through the electrolyte composition of the carrier solution. Albumin 4% solutions generally exert more modest Atot-related effects than hyperoncotic preparations, although the overall physicochemical response depends on the composition of the carrier vehicle [5]. Since commercially available albumin preparations differ substantially in sodium concentration, chloride concentration, stabilizers, pH, osmolality, and effective SID, their acid–base effects cannot be generalized. Consequently, post-infusion acid–base changes should always be interpreted in relation to the specific formulation administered rather than assuming a uniform effect across all albumin products.

This formulation-dependent balance has also been demonstrated in human studies. In a randomized physiological study, Bruegger et al. showed that infusion of 20% albumin increased both the strong ion difference and the concentration of weak plasma acids, indicating that the observed acid–base response reflects the net effect of these opposing physicochemical mechanisms rather than a uniformly acidifying action of albumin [40].

From a clinical perspective, these differences are particularly relevant in septic shock, where hypoalbuminemia and cumulative chloride exposure frequently coexist. Under these conditions, isolated interpretation of bicarbonate concentration or base excess may incompletely capture the complexity of the underlying acid–base disorder. The expected directional acid–base effects of common resuscitation fluids are summarized in Table 2.

## 10. Masked Acidosis and Bedside Interpretation Pitfalls

**Figure 3:**
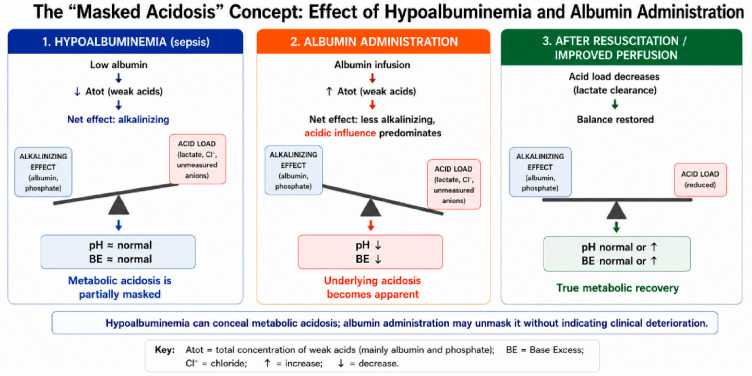
Schematic representation of the “masked acidosis” concept during hypoalbuminemia and after albumin administration. Illustrative conceptual simulations based on simplified Stewart principles; values are intended to demonstrate directional trends rather than calibrated physiological predictions.

Hypoalbuminemia is common in sepsis and septic shock. From a Stewart perspective, reduced albumin concentration lowers Atot and therefore exerts an alkalinizing tendency, partially offsetting the acidifying effects of hyperchloremia, hyperlactatemia, or unmeasured anions. Consequently, apparently near-normal pH or base excess values may conceal clinically relevant metabolic derangement rather than exclude metabolic acidosis [41]. This mechanism is illustrated schematically in Figure 3.

Albumin administration may partially reverse this masking effect by restoring weak acid concentration, producing a modest reduction in pH or base excess despite stable or improving hemodynamics and tissue perfusion [29,42,43]. Post-infusion acid–base changes should be interpreted together with lactate, chloride concentration, serum albumin, renal function, and hemodynamic trends rather than in isolation.

Saline and albumin may produce similar arterial blood gas patterns through different physicochemical mechanisms. Saline-associated acidosis is predominantly mediated by chloride-induced SID reduction, whereas albumin-associated acidification mainly reflects an increase in Atot. Similar pH or base excess values may therefore represent distinct physiological states with different implications for fluid therapy.

Three practical pitfalls should be avoided. First, near-normal pH or base excess should not be considered reassuring when severe hypoalbuminemia coexists with elevated lactate, hyperchloremia, renal dysfunction, or unmeasured anions. Second, a modest fall in pH after albumin administration should not be interpreted as worsening tissue hypoperfusion without considering concurrent hemodynamic and perfusion trends [44]. Third, chloride-associated acidosis should not be considered equivalent to lactate-associated acidosis, because the former is primarily SID-mediated whereas the latter usually reflects altered metabolism, impaired clearance, or tissue hypoperfusion. Representative clinical scenarios are summarized in Table 3.

## 11. Integrated Bedside Interpretation

**Figure 4:**
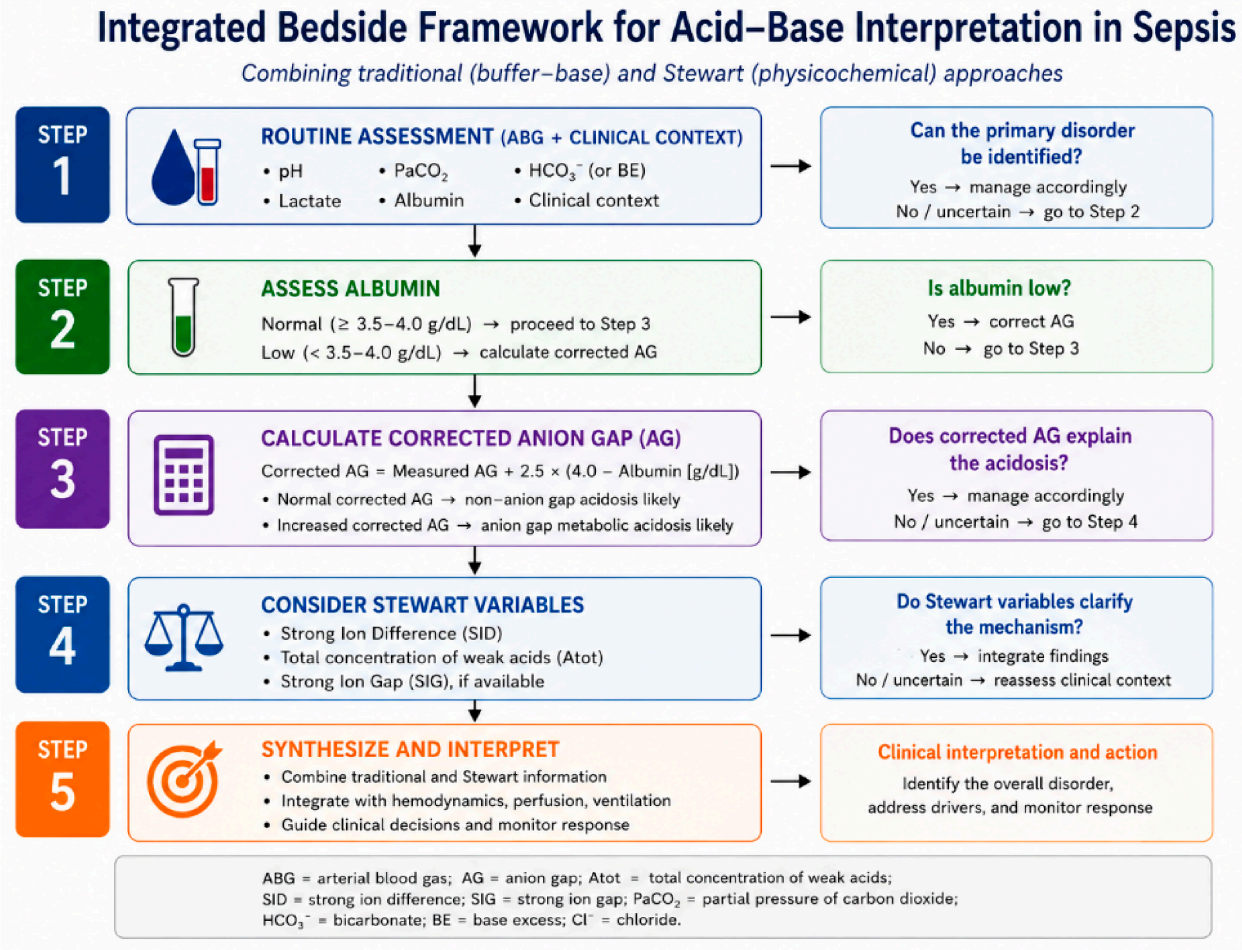
Integrated bedside framework combining traditional and Stewart variables for interpretation of acid–base disorders during albumin administration in sepsis.

A practical stepwise approach may facilitate bedside interpretation. First, conventional arterial blood gas analysis (pH, PaCO_2_, bicarbonate, and base excess), together with serum lactate and albumin concentration, should be used to identify the primary acid–base disturbance. Second, albumin-corrected AG may provide additional information in hypoalbuminemic patients, although it should be interpreted as an approximate bedside tool rather than a definitive measure of unmeasured anions. Third, when acid–base abnormalities remain incompletely explained, Stewart-derived variables such as SID and, where available, the strong ion gap (SIG) may provide additional mechanistic insight. This integrated approach helps distinguish SID-mediated acidosis from Atot-mediated acidification, identify masked metabolic acidosis, and avoid overinterpreting isolated changes in bicarbonate or base excess [29,30,44]. A proposed bedside framework is shown in Figure 4.

Traditional variables remain essential for rapid assessment of acidemia, respiratory compensation, and the metabolic component of acid–base disturbance. Stewart-derived variables add mechanistic resolution when hypoalbuminemia, chloride exposure, renal dysfunction, or mixed metabolic disorders coexist. In this context, albumin concentration, corrected anion gap, chloride, SID, and unmeasured ions may clarify apparently paradoxical arterial blood gas findings.

Acid–base interpretation should remain linked to organ-level physiology [45,46]. Lactate kinetics, vasopressor requirements, urine output, fluid balance, renal function, respiratory status, and overall perfusion should determine whether a change in pH reflects true clinical deterioration or a predictable physicochemical shift. A suggested integrated bedside interpretation framework is summarized in Table 4.

## 12. Cardiorespiratory Interactions During Albumin Administration and Acid–Base Resuscitation

**Figure 5:**
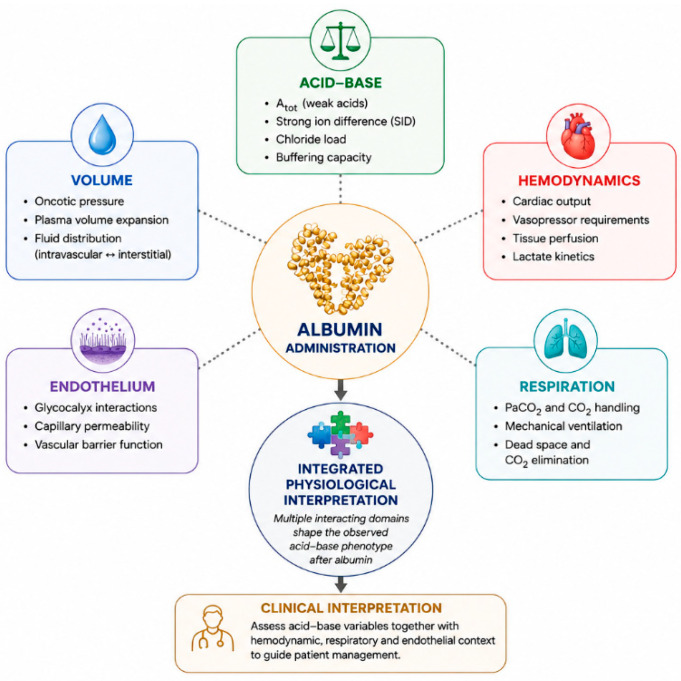
Cardiorespiratory interactions during albumin administration in septic shock integrating acid–base physiology, respiratory mechanics, and hemodynamic response. Multiple arrows a greater magnitude of effect.

Acid–base effects of albumin should be interpreted within the cardiorespiratory context of sepsis. In mechanically ventilated patients, changes in PaCO_2_, ventilation, and tissue perfusion may substantially influence their clinical expression. The major physiological interactions influencing albumin-related acid–base changes are summarized in Figure 5.

Carbon dioxide represents the respiratory determinant of pH within both traditional and physicochemical frameworks [47]. Therefore, ventilatory settings and CO_2_ clearance may interact with SID- and Atot-mediated metabolic effects, particularly in patients managed with protective ventilation or permissive hypercapnia [48,49].

Albumin may also influence respiratory physiology through changes in oncotic pressure, endothelial permeability, and pulmonary fluid distribution. Under selected conditions, restoration of oncotic pressure may favor reabsorption of extravascular lung water, although these effects are context-dependent and should not be interpreted as evidence that albumin uniformly improves oxygenation or respiratory outcomes [50,51].

Correction of effective hypovolemia may improve cardiac output, tissue perfusion, and oxygen delivery, even when arterial pH decreases modestly because of increased Atot [52,53,54]. Conversely, hyperchloremic acidosis and severe acidemia may adversely affect cardiovascular function and vasomotor responsiveness [55].

Positive-pressure ventilation further modifies this balance through its effects on venous return and CO_2_ elimination [50,51]. In septic patients with ARDS, the coexistence of permissive hypercapnia, chloride-rich fluid exposure, hypoalbuminemia, and lactate accumulation may generate mixed acid-base disorders in which apparently small pH changes conceal substantial physiological heterogeneity [53,54].

## 13. Randomized Controlled Trials: Clinical Context for Albumin and Fluid Composition

Although this review focuses on acid–base physiology, interpretation of albumin therapy in sepsis should be framed within the evidence provided by major randomized clinical trials evaluating albumin, colloids, saline, and balanced crystalloids in critically ill patients. The major randomized trials should be interpreted considering current sepsis guidelines and contemporary fluid resuscitation practice. However, these recommendations are based primarily on clinical outcomes rather than on acid–base physiology. None of the major trials were specifically designed to investigate Stewart variables, strong ion difference (SID), total weak acids (Atot), or chloride-mediated acid–base mechanisms.

The SAFE trial compared 4% albumin with normal saline in 6997 critically ill patients requiring fluid resuscitation and found no difference in 28-day mortality [4]. From a physiological perspective, SAFE is particularly informative because it directly compared two fluids with markedly different physicochemical properties. Albumin increases plasma weak-acid concentration, whereas normal saline has an effective SID close to zero and may promote hyperchloremic metabolic acidosis. Nevertheless, acid–base variables were not prospectively evaluated, precluding assessment of whether these mechanistic differences influenced clinical outcomes.

The ALBIOS trial randomized 1818 patients with severe sepsis or septic shock to receive 20% albumin plus crystalloids or crystalloids alone, targeting serum albumin concentrations ≥ 30 g/L [5]. Although albumin administration improved hemodynamic variables and influenced cumulative fluid balance, no significant reduction in 28- or 90-day mortality was observed in the overall population. ALBIOS did not evaluate Stewart-derived variables, albumin-corrected acid–base parameters, or physicochemical mechanisms, limiting mechanistic interpretation of the observed physiological effects.

The CRISTAL trial compared colloids with crystalloids in ICU patients with hypovolemic shock [56]. Because albumin represented only one component of the colloid arm, the study cannot be interpreted as albumin-specific evidence. Nonetheless, it provides useful context by illustrating that differences in resuscitation fluids do not necessarily translate into consistent survival benefits despite distinct physiological properties.

More recently, the ARISS trial evaluated albumin replacement in patients with septic shock [6]. Albumin administration appeared safe but did not improve 90-day survival. The trial was terminated prematurely, limiting statistical power and the certainty of its conclusions. In addition, a subsequent correction clarified aspects of the lactate inclusion criterion reported in Section 2. Like previous trials, ARISS was not designed to evaluate acid–base endpoints or physicochemical mechanisms, and therefore provides limited insight into the relationship between albumin administration, Atot, SID, and acid–base equilibrium.

The ICARUS-ED pilot trial evaluated early albumin administration in septic patients presenting to the emergency department [43]. Although albumin did not significantly improve the primary endpoint of systolic blood pressure at 24 h, it was associated with reduced cumulative fluid administration, lower vasopressor requirements, and favorable signals in organ dysfunction. Given its pilot design and limited sample size, these findings should be considered hypothesis-generating rather than evidence of clinical benefit.

The major randomized trials evaluating albumin or colloids in critical illness are summarized in Table 5.

Complementary evidence regarding chloride balance and SID derives from trials comparing balanced crystalloids with normal saline. SPLIT showed no significant reduction in acute kidney injury with Plasma-Lyte compared with saline [30]. SMART demonstrated fewer major adverse kidney events among critically ill adults receiving balanced crystalloids [35], whereas BaSICS and PLUS failed to demonstrate consistent improvements in mortality or renal outcomes [37,38]. Similarly, CLASSIC and CLOVERS, although designed to evaluate fluid strategy rather than fluid composition, highlighted that differences in resuscitation approach do not necessarily translate into improved survival despite potentially distinct physiological consequences. The major randomized trials comparing balanced crystalloids and saline are summarized in Table 6.

Collectively, these studies suggest that predictable physicochemical effects do not invariably translate into measurable patient-centered benefits. Albumin administration may increase Atot, modify bicarbonate concentration, base excess, and anion-gap interpretation, whereas balanced crystalloids may reduce chloride exposure and preserve SID. However, the clinical significance of these mechanistic effects remains uncertain, reinforcing the distinction between physiological acid–base responses and hard clinical outcomes.

## 14. Future Directions

Future research should move beyond simplistic classifications of fluids as “acidifying” or “alkalinizing.” The physiological effects of albumin likely depend on dynamic interactions among Atot, SID, chloride balance, dilution, renal compensation, and tissue perfusion.

Prospective physiological studies comparing albumin 4%, albumin 20%, balanced crystalloids, and saline-based strategies using both traditional and Stewart metrics may clarify these interactions.

Integration of physicochemical analyses into future sepsis trials may also improve interpretation of arterial blood gas trajectories during resuscitation.

## 15. Limitations

This review has some limitations. First, many physicochemical interpretations discussed herein derive from conceptual and mechanistic models rather than from randomized physiological trials specifically designed around Stewart variables. Second, the conceptual simulations presented are illustrative and are not intended as calibrated patient-level predictions. The conceptual simulations were developed as educational tools to facilitate understanding of physicochemical principles and have not been prospectively or experimentally validated. Furthermore, commercial albumin formulations differ substantially in electrolyte composition and buffering characteristics, limiting the generalizability of any formulation-independent representation. Third, acid–base responses during sepsis are highly dynamic and influenced by renal compensation, respiratory status, vasopressor exposure, and fluid balance, factors that cannot be fully captured by simplified frameworks alone. Finally, the integrated bedside framework proposed in this review reflects the authors’ synthesis of the available physiological and clinical evidence and has not been prospectively validated as a clinical decision-support tool.

## 16. Conclusions

Albumin affects acid–base equilibrium through weak acid expansion, chloride balance, dilution, and strong ion physiology. In septic patients, particularly those with hypoalbuminemia or chloride exposure, traditional and Stewart approaches should be integrated rather than opposed. This combined interpretation may help clinicians distinguish true metabolic deterioration from predictable physicochemical changes after albumin administration. Integrating traditional and physicochemical frameworks does not replace clinical judgment but provides a more comprehensive physiological basis for interpreting acid–base changes during fluid resuscitation in sepsis.

## Figures and Tables

**Table 1:** Comparison between traditional and Stewart approaches to albumin-related acid–base physiology. In the Stewart framework, a decrease in the total concentration of non-volatile weak acids (Atot) shifts acid–base balance toward alkalosis, whereas an increase in Atot shifts it toward acidosis.

Feature	Traditional Approach	Stewart Approach
Main determinant of pH	HCO_3_^−^/PaCO_2_ ratio	SID, Atot, PaCO_2_
Role of albumin	Non-bicarbonate buffer	Weak non-volatile acid
Key variables	HCO_3_^−^, BE, AG	SID, Atot, SIG
Hypoalbuminemia	Reduced buffering; lower measured AG	↓ Atot → alkalinizing tendency
Albumin administration	Restores buffer base; may reduce BE	↑ Atot → acidifying tendency
Saline acidosis	Hyperchloremic/dilutional acidosis	↓ SID acidosis
Clinical strength	Simplicity and bedside familiarity	Mechanistic explanation of mixed disorders
Main limitation	Limited mechanistic resolution in complex ICU states	Less intuitive and more calculation-dependent

**Table 2:** Conceptual acid–base effects of common resuscitation fluids.

Fluid	Typical Carrier Composition	Typical Effective SID	Weak Acid Effect	Expected Net Effect
Normal saline	154 Na/154 Cl	~0	None	SID-mediated metabolic acidosis
Lactated Ringer	balanced electrolyte solution	~28	None	Near-neutral/slightly alkalinizing
Albumin 4%	formulation-dependent	formulation-dependent	Mild ↑ Atot	depends on carrier + albumin concentration
Albumin 20%	formulation-dependent	formulation-dependent	Marked ↑ Atot	balance between Atot and carrier composition

Values are conceptual and formulation-dependent; commercial albumin preparations differ in electrolyte composition, stabilizers, osmolality, pH and effective SID. Values vary according to sodium/chloride content and stabilizers of commercial preparations (e.g., Alburex^®^, Albuminar^®^, CSL Behring formulations (King of Prussia, PA, USA)). Upward arrows indicate an increase.

**Table 3:** Representative clinical scenarios interpreted through traditional and Stewart frameworks. Downward arrows a decrease.

Scenario	Traditional Interpretation	Stewart Interpretation
Hypoalbuminemia + high lactate	Mild metabolic acidosis	Masked acidosis
Albumin infusion + ↓ pH	Worsening metabolic acidosis	Restoration of Atot
Hyperchloremia	Hyperchloremic acidosis	↓ SID acidosis
Near-normal BE with low albumin	Mild derangement	Hidden metabolic abnormality

**Table 4:** Suggested integrated bedside interpretation framework.

Variable	Physiological Meaning
pH	Overall acidemia/alkalemia
HCO_3_^−^	Metabolic component
Base excess	Buffer balance
Lactate	Tissue hypoperfusion, impaired clearance, or altered cellular metabolism
Chloride	SID effect
Albumin	Atot
Corrected anion gap	Hidden anions
SID	Physicochemical balance

**Table 5:** Major randomized controlled trials evaluating albumin therapy in critically ill patients.

Trial	Year	Population	Intervention	Comparator	Principal Finding	Relevance to This Review
SAFE	2004	Critically ill patients requiring fluid resuscitation	4% albumin	Normal saline	No difference in 28-day mortality	Largest albumin-versus-saline comparison; acid–base mechanisms were not evaluated
CRISTAL	2013	ICU patients with hypovolemic shock	Colloids (including albumin)	Crystalloids	No significant difference in 28-day mortality	Provides contextual evidence for colloids but is not albumin-specific
ALBIOS	2014	Severe sepsis or septic shock	20% albumin plus crystalloids targeting serum albumin ≥ 30 g/L	Crystalloids alone	No reduction in 28- or 90-day mortality overall; improved hemodynamic variables	Most relevant sepsis-specific albumin trial; did not evaluate Stewart variables or acid–base endpoints
ICARUS-ED	2025	Emergency department patients with sepsis	Early albumin administration	Standard fluid resuscitation	No improvement in the primary endpoint; favorable physiological signals	Pilot study; hypothesis-generating and not powered for clinical outcomes
ARISS	2026	Septic shock	Albumin replacement targeting serum albumin concentration	Standard fluid therapy	No improvement in 90-day mortality; trial terminated prematurely	Contemporary septic shock trial; limited by early termination and no dedicated acid–base evaluation

**Table 6:** Major randomized controlled trials comparing balanced crystalloids and normal saline.

Trial	Year	Population	Intervention	Comparator	Principal Finding	Relevance to This Review
SPLIT	2015	ICU patients	Plasma-Lyte 148	Normal saline	No significant difference in acute kidney injury	Initial balanced crystalloid trial; limited chloride exposure difference
SMART	2018	Critically ill adults	Balanced crystalloids	Normal saline	Lower incidence of major adverse kidney events	Landmark trial supporting the physiological relevance of chloride load and strong ion difference
SALT-ED	2018	Non-ICU adults treated in the emergency department	Balanced crystalloids	Normal saline	Lower incidence of major adverse kidney events	Extended balanced crystalloid evidence beyond the ICU
BaSICS	2021	Critically ill adults	Plasma-Lyte 148	Normal saline	No difference in 90-day mortality	Suggested that physiological advantages of balanced solutions may not consistently translate into survival benefits
PLUS	2022	ICU patients	Plasma-Lyte 148	Normal saline	No significant difference in mortality or acute kidney injury	Contemporary multicenter trial providing context for chloride- and SID-related physiology

## Data Availability

No new data were created or analyzed in this study. Data sharing is not applicable to this article.

## Supplementary Materials

The following supporting information can be downloaded at: https://www.mdpi.com/article/10.3390/jcm15155789/s1, Supplementary Methods: Conceptual Physicochemical Simulations; Table S1: Assumptions underlying the conceptual simulations; Table S2: Physicochemical variables considered in the conceptual simulations; Figure S1: Conceptual framework used to generate the physicochemical simulations.

## Abbreviations

The following abbreviations are used in this manuscript:

AG	Anion gap
AKI	Acute kidney injury
Atot	Total concentration of weak acids
BE	Base excess
ICU	Intensive care unit
SID	Strong ion difference
SIG	Strong ion gap
