# Supplementary material for: The Acid–Base Effects of Albumin in Sepsis: Reconciling the Stewart Physicochemical Approach with the Traditional Buffer–Base Paradigm
Source: J Clin Med. 2026 Jul 24;15(15):5789. doi: 10.3390/jcm15155789 (PMC13467214; doi:10.3390/jcm15155789)

Supplementary Materials

The Acid–Base Effects of Albumin in Sepsis: Reconciling the Stewart Physicochemical Approach with the Traditional Buffer–Base Paradigm

Daniele Orso, Raffaele Saro, Giorgio Della Rocca

1. Supplementary Methods

Conceptual Physicochemical Simulations

The conceptual simulations presented in Figures 2 and 3 were developed exclusively for educational purposes to illustrate the expected direction of acid–base changes according to Stewart's physicochemical framework. They were designed as mechanistic illustrations rather than quantitative physiological models and therefore should not be interpreted as patient-specific predictions.

A simplified theoretical plasma compartment was assumed under steady-state conditions. Respiratory status ($PaCO_2$) was considered constant throughout the simulations, and no dynamic changes in ventilation, renal compensation, endogenous acid production, tissue perfusion, capillary leak, or fluid redistribution were modeled. Each fluid was evaluated as an isolated intervention to demonstrate its expected physicochemical effects under controlled conditions.

The simulations were based on established Stewart principles describing acid–base equilibrium as a function of three independent variables:

- Partial pressure of carbon dioxide ($PaCO_2$)
- Strong ion difference (SID)
- Total concentration of weak acids (Atot)

For each fluid, the expected direction of change in SID, Atot, and acid–base equilibrium was inferred qualitatively from its physicochemical characteristics.

For albumin-containing solutions, two concurrent mechanisms were considered:

1. Expansion of the plasma weak-acid pool (increase in Atot) following albumin administration.
2. The electrolyte composition of the carrier solution, particularly its sodium and chloride concentrations, influencing the effective strong ion difference.

For crystalloid solutions, only changes related to electrolyte composition and SID were considered.

No attempt was made to estimate the magnitude of pH variation because this depends on numerous physicochemical variables—including respiratory compensation, renal adaptation, ongoing lactate

production, plasma protein concentration, and the physicochemical characteristics of the specific commercial preparation—which were intentionally not modeled.

Consequently, Figures 2 and 3 depict only qualitative trends (increase, decrease, or no major change) rather than validated quantitative estimates.

Commercial Albumin Preparations

Commercial human albumin preparations differ considerably with respect to:

- sodium concentration;
- chloride concentration;
- stabilizing agents (e.g., sodium caprylate and N-acetyl-DL-tryptophanate);
- pH;
- osmolality;
- carrier solution composition.

These differences influence the effective SID of the infused solution and therefore may modify the overall acid–base response. Accordingly, the conceptual simulations presented in this review should be interpreted as illustrating general physiological mechanisms rather than predicting the effects of any specific commercial formulation.

2. Supplementary Table S1

Assumptions underlying the conceptual simulations

Variable	Assumption
Purpose	Educational illustration of Stewart physicochemical principles
Model	Simplified theoretical plasma compartment
Respiratory status	Constant $PaCO_2$
Ventilatory compensation	Not modeled
Renal compensation	Not modeled
Lactate kinetics	Assumed stable (no dynamic production or clearance)
Tissue perfusion	Not modeled
Fluid redistribution	Not modeled
Capillary leak	Not modeled
Commercial albumin formulations	Considered formulation-dependent; no specific manufacturer modeled
Outputs	Direction of change in SID, Atot, and acid–base equilibrium only
Intended interpretation	Mechanistic illustration; not a quantitative physiological prediction

3. Supplementary Table S2

Physicochemical variables considered in the conceptual simulations

Variable	Physiological role
$PaCO_2$	Independent respiratory determinant of acid–base equilibrium
SID	Principal physicochemical determinant related to strong ions
Atot	Total concentration of weak acids, predominantly albumin and phosphate
Albumin	Major plasma weak acid contributing to Atot
Chloride	Principal determinant of SID changes during fluid therapy
Lactate	Strong anion contributing to reductions in SID

Supplementary Notes

The conceptual simulations were developed solely to facilitate interpretation of the physiological mechanisms discussed throughout this review. They are not intended to replace patient-specific acid–base analysis or validated quantitative physicochemical modeling.

The figures should therefore be interpreted as educational schematics illustrating the expected direction of acid–base changes under simplified conditions. Clinical acid–base responses remain dependent on patient-specific physiological factors, including respiratory compensation, renal function, baseline hypoalbuminemia, lactate kinetics, the volume administered, and the composition of the specific resuscitation fluid.

4. Supplementary Figure S1

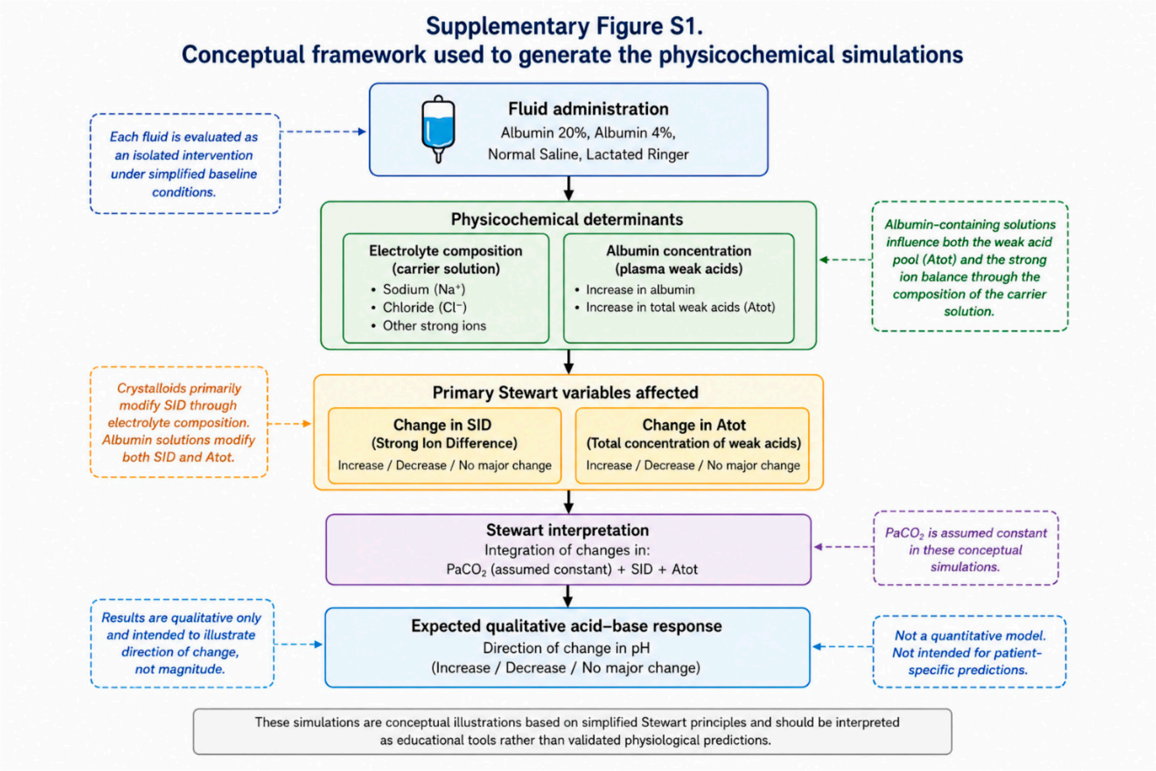

Supplement: Supplementary file 1 [file jcm-15-05789-s001.zip › jcm-4429492-supplementary.pdf]
